# Utility value estimates in cardiovascular disease and the effect of changing elicitation methods: a systematic literature review

**DOI:** 10.1186/s12955-020-01407-y

**Published:** 2020-07-27

**Authors:** Marissa Blieden Betts, Pratik Rane, Evelien Bergrath, Madhura Chitnis, Mohit Kumar Bhutani, Claudia Gulea, Yi Qian, Guillermo Villa

**Affiliations:** 1 Evidence Synthesis, Modeling & Communication Evidera Inc, Waltham, MA USA; 2 Amgen Inc, Global Health Economics, Thousand Oaks, CA USA; 3 BresMed Health Solutions Ltd., Gurugram, Haryana India; 4 Evidence Synthesis, Modeling & Communication, Evidera, London, UK; 5 Amgen Inc, Intercontinental HQ-Value, Access & Policy, Thousand Oaks, CA USA; 6 Amgen (Europe) GmbH, Global Health Economics, Zug, Switzerland

**Keywords:** Cardiovascular disease, EQ-5D, Health state utilities, Trends

## Abstract

**Objective:**

Identify the most recent utility value estimates for cardiovascular disease (CVD) via systematic literature review (SLR) and explore trends in utility elicitation methods in the last 6 years.

**Methods:**

This SLR was updated on January 25, 2018, and identified studies reporting utilities for myocardial infarction (MI), stroke, angina, peripheral artery disease (PAD), and any-cause revascularization by searching Embase, PubMed, Health Technology Assessment Database, and grey literature.

**Results:**

A total of 375 studies reported CVD utilities (pre-2013 vs post-2013: MI, 38 vs 32; stroke, 86 vs 113; stable angina, 8 vs 9; undefined/unstable angina, 23 vs 8; PAD, 29 vs 13; revascularization, 54 vs 40). Median average utilities for MI, stroke, and revascularization increased over time (pre-2013 vs post-2013: MI, 0.71 vs 0.79; stroke, 0.63 vs 0.64; revascularization, 0.76 vs 0.81); angina and PAD showed a decrease in median values over time (stable angina, 0.83 vs 0.72; undefined/unstable angina, 0.70 vs 0.69; PAD, 0.76 vs 0.71). The proportion of utility estimates from trials increased across health states (pre-2013 vs post-2013: 22.5% vs 37.2%), as did the proportion of trials using the EuroQol Five Dimensions Questionnaire (EQ-5D; pre-2013 vs post-2013: 73.8% vs 91.4%). Use of methods such as the standard gamble, time trade-off, and Health Utilities Index has declined.

**Conclusions:**

Health state utilities for cardiovascular health states have changed in the last 6 years, likely due to changes in the types of studies conducted, the patient populations evaluated, and possibly changing utility elicitation methods. The EQ-5D has been used more frequently.

## Introduction

Cardiovascular disease (CVD) is the leading cause of mortality worldwide and imposes a significant clinical and economic burden on society. In 2016, CVD accounted for approximately 17.9 million deaths worldwide, representing 31% of all global cases [1]. In 2010, the estimated global cost of CVD was $863 billion, and it is estimated to rise to $1044 billion by 2030 [2, 3]. CVD causes long-term disability, affecting the health-related quality of life (HRQoL) of patients [4].

Although conventional treatments reduce the risk of CVD, exploring new drugs that provide clinical and economic value—given the current clinical and economic burden—is an ongoing need. Economic evaluations, such as cost-effectiveness analyses (CEAs), are important for comparing new and existing treatments; they are used to determine a treatment’s economic value and to demonstrate that value to patients, physicians, and third-party payers [5, 6]. These analyses often inform healthcare reimbursement decisions [7]. The preferred outcome measure of one type of CEA—cost-utility analyses—is quality-adjusted life-years (QALYs) gained, where the quality of life adjustment is based on utility values [8]. In order to generate QALYs, health utilities are constructed with values that are usually anchored at 0 and 1, which represent the strength of preferences for health states (ie, 1 represents full health and 0 represents dead). Some methods allow for health states to be regarded as worse than death and have negative valuations [9, 10].

Health state utilities are generated using direct methods, indirect methods, or a combination of the two. The main difference between these methods is that direct methods are used to elicit patients’ preferences to health states, whereas indirect methods evaluate patients’ current quality of life and apply population preferences to weight these scores to obtain a utility estimate [4, 11].

Smith et al. 2013 previously conducted a systematic literature review (SLR) in 2012 (including studies published between September 1992 and September 2012) that found utility values were lower in patients who experienced cardiovascular (CV) events than in patients who did not [12]. Furthermore, the authors suggested that the utility estimates for each individual CV event varied greatly, likely due to differences in assessment methodologies and patient populations. The goal of this current systematic review was to update and expand upon the review by Smith et al., which evaluated utilities for myocardial infarction (MI), angina, and stroke to identify the most recent utility values for these health states, as well as revascularization and peripheral artery disease (PAD) and to gain insight into changing trends in utility elicitation methods and values, which can be used to inform/calculate QALYs. In addition, this review identified the methods used to elicit utilities and examine variability among utility values for a given CV health state, and how those values may be impacted by factors such as type of respondent, study design, and geographic location.

## Methods

### Search strategy

The methodology of this SLR update was consistent with the original SLR presented in Smith et al. [12]. The SLR was designed in accordance with the Preferred Reporting Items for Systematic Reviews and Meta-Analyses (PRISMA) standards. The original search identified studies published between September 1992 and September 2012. For the update, an electronic database search was conducted in Embase, MEDLINE, and Health Technology Assessment Database (HTAD), and it used search algorithms that were aimed at identifying relevant studies published between September 2012 and January 2018 presenting utilities for MI, stroke, and angina, as well as studies published between September 1992 and January 2018 presenting utilities for new CV health states, PAD, and revascularization (any-cause, including both peripheral and coronary). The search identified publications using keywords related to the CV health states (MI, stroke, angina, PAD, and revascularization) and utilities (eg, utility, time trade-off [TTO], standard gamble [SG], Health Utilities Index [HUI], Short-Form Six Dimensions [SF-6D], and EuroQol Five Dimensions Questionnaire [EQ-5D]). These most commonly used direct and indirect methods across the CV events of interest are described in Table 1. The complete list of keywords is provided in Supplementary Tables 1, 2, and 3. Only papers published in English, pertaining to humans, and indexed during the search period (September 1992 to January 2018) were eligible for inclusion. To supplement the database searches, grey literature (reports and conference abstracts) presented by relevant scientific organizations or health technology assessment (HTA) body websites within the last 6 years (2012–2018) were also searched using the same main keywords. Supplementary Table 4 provides an overview of the scientific conferences and HTA websites that were examined for this review.

**Table 1:** Common health state utility elicitation methods

Type of methods	Common approaches or tools	Description of methods
Direct methods	• SG • TTO	These are interview-based and used to capture values that patients or the general public assign to a health state [13]. During the interviews, individuals (patients or members of the general public) identify their preferences for either their current health or scenarios (also called vignettes) that describe various health states by engaging in choice-based tasks [4].
Indirect methods	• HUI mark 2 and 3 • EQ-5D • SF-6D	These questionnaires typically evaluate domains such as disability, mental health, and pain. Responses are converted to utilities by means of “tariffs” or “weights.” Published tariffs are used to weigh the scores of each domain based on the importance of that domain to that population or country. Tariffs are available as a result of separate and previous exercises in which various possible health states have been calibrated by means of a trade-off, SG, or well-known preference-based methods, such as EQ-5D, HUI mark 2, and SF-6D, from a sample of the general population [14]. The indirect measures differ in what dimensions their questionnaires include, how many response levels each question has, and the direct valuation method used to create the tariff.

*Abbreviations: EQ-5D* EuroQol Five Dimensions Questionnaire, *HUI* Health Utilities Index, *SG* Standard gamble, *SF-6D* Short-Form Six Dimensions, *TTO* Time trade-off

To understand the changing trends in utility value estimates (median and interquartile ranges [IQRs]) and methods used for utility elicitation, studies from the previous review and updated SLR were compared. This was done by assessing trends in the last 6 years by stratifying the period as pre-2013 (1992–2012) vs post-2013 (2013–2018).

### Study review and selection

All abstracts were manually reviewed by a single reviewer, who used prespecified inclusion and exclusion criteria (Supplementary Table 5; participants, interventions, comparators, outcomes, and study design [PICOS] criteria) to select primary studies and systematic reviews that reported utilities for CV health states. All papers accepted during abstract screening were reviewed in full text by 2 independent reviewers, who also used the same prespecified inclusion and exclusion criteria. Any discrepancies in the decisions were reviewed and resolved by a third, senior reviewer.

This review was not limited by the type of utility measure; however, simple visual analogue scale (VAS) methods, which represent a direct approach, were not considered as valid utility elicitation methods [15] and were excluded.

### Data extraction

The following data and characteristics were captured from all articles included in the systematic review: publication year, study design, interventions (if applicable), country, CV health state, utility methods, utility values, population of respondents, and sample size. The studies on angina that did not specify whether the patients had stable or unstable angina, or reported on a mixed angina population, were grouped with the unstable angina studies. This was because the utility values for unspecified and unstable angina were similar to each other (average of 0.71 for both), whereas those for stable angina appeared to be slightly higher (average of 0.77).

### Qualitative synthesis

Studies that were published before 2013 (1992–2012) were compared with studies published after 2013 (2013–2018) in terms of utility value estimates and utility elicitation methods in order to explore trends over time by means of a qualitative synthesis. As only a qualitative synthesis was prospectively planned, no statistical inference was performed. Additionally, due to the wide range and skewed utility value distributions in the identified studies, median and IQRs were generated from average utility values reported (as either mean or median) in the literature for both time periods.

### Definition of minimally important differences

Although HRQoL is currently recognized as an important endpoint in clinical trials, the meaningfulness of HRQoL scores may not be apparent to patients, clinicians, or researchers [16]. Minimally important differences (MIDs) for health state utilities vary by measure and are not well established. It has been suggested that differences among health state utilities of at least 0.05 can be considered clinically important [17].

## Results

**Fig. 1:**
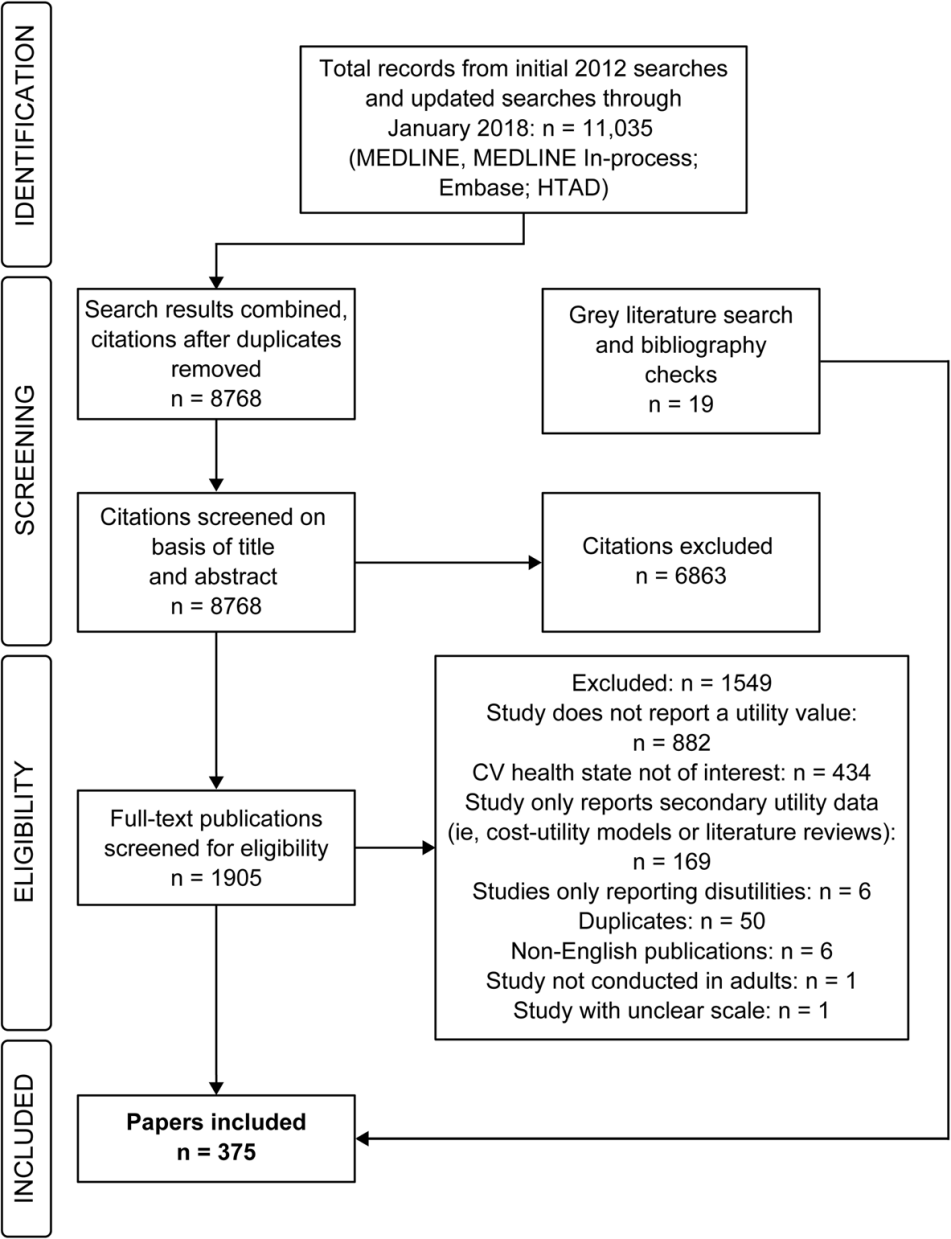
PRISMA diagram. *Abbreviations: CV* cardiovascular, *HTAD* Health Technology Assessment Database

A total of 11,035 citations were identified across the databases. After removing duplicates, 8768 unique citations were eligible for abstract screening. Of these, 1905 were included for full-text screening, during which 1549 articles further were excluded, as described in the PRISMA diagram (Fig. 1). In total, 375 publications reported qualitative and quantitative utility values in MI, stroke, angina, revascularization, and/or PAD and were included in the SLR.

Among the included papers, 70 publications reported utilities associated with MI, of which 38 were published before 2013 and 32 after 2013. For stroke, a total of 199 publications reporting utilities were identified, of which 86 were published before 2013 and 113 after 2013. Additionally, 17 publications reported stable angina utilities (8 pre-2013 and 9 post-2013), 31 publications reported utilities for undefined/unstable angina (23 pre-2013 and 8 post-2013), 42 publications for PAD (29 pre-2013 and 13 post-2013), and 94 publications for revascularization (54 pre-2013 and 40 post-2013). The process and results of the searching and screening are presented in Fig. 1*.*

### Trends in utility values over time by type of CV event

**Fig. 2:**
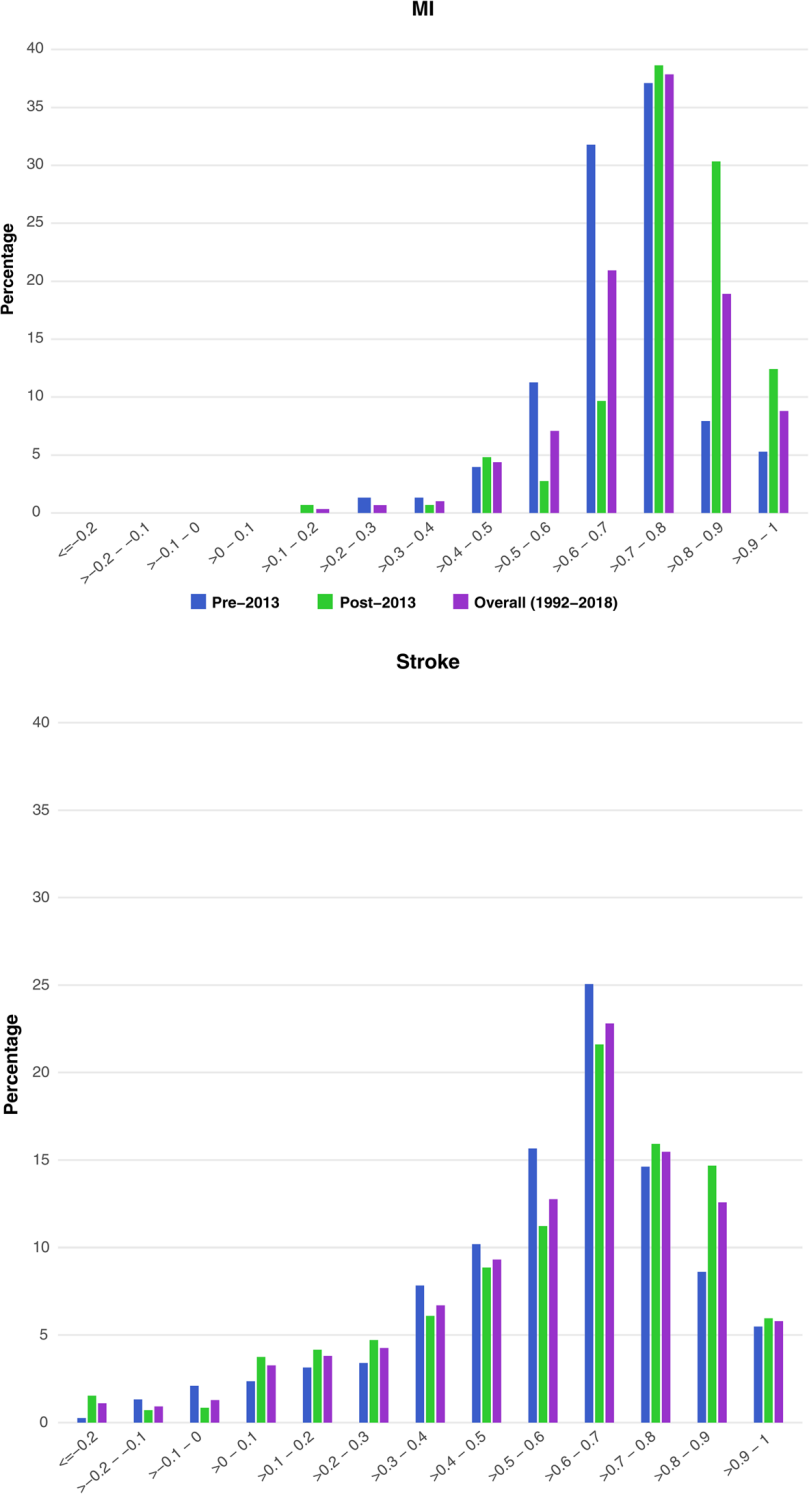
Distribution of utility values by event type (MI, stroke)**.***Abbreviation: MI* myocardial infarction

Table 2 presents the estimated median (IQR) utility values by method of elicitation, type of disease, and publication year. When looking across CV events, regardless of the utility measure used, the median utility values were lowest for stroke compared with the other CV events of interest. When comparing median (IQR) utility values between the 2 time periods (pre-2013 vs post-2013), several trends were observed: median (IQR) utility values for MI and revascularization showed an increase over time (pre-2013 vs post-2013, respectively: MI, 0.71 [0.64–0.76] vs 0.79 [0.73–0.86]; revascularization, 0.76 [0.67–0.82] vs 0.81 [0.74–0.85]), and values for stroke showed a slight increase over time (0.63 [0.45–0.72] vs 0.64 [0.44–0.78]). However, values for stable angina (0.83 [0.68–0.94] vs 0.72 [0.66–0.78]), unstable/unspecified angina (0.70 [0.62–0.82] vs 0.69 [0.62–0.85]), and PAD (0.76 [0.69–0.85] vs 0.71 [0.63–0.78]) decreased over time. It should be noted that only 4 studies reported utilities for unstable angina, whereas the majority of studies in this group did not specify the type of angina. Figure 2 presents the distribution of utility values for MI and stroke. Most studies reported utilities between > 0.7 and 0.8 for MI in both time periods and values from > 0.6 to 0.7 for stroke.

**Table 2:** Estimated median (IQR) average utility values by method, type of disease, and publication year

	MI		Stroke		Stable angina		Unstable/unspecified angina^**a**^		PAD		Revascularization	
	Pre-2013 ***n*** = 38	Post-2013 ***n*** = 32	Pre-2013 ***n*** = 86	Post-2013 ***n*** = 113	Pre-2013 ***n*** = 8	Post-2013 ***n*** = 9	Pre-2013 ***n*** = 23	Post-2013 ***n*** = 8	Pre-2013 ***n*** = 29	Post-2013 ***n*** = 13	Pre-2013 ***n*** = 54	Post-2013 ***n*** = 40
Total	0.71 (0.64–0.76)	0.79 (0.73–0.86)	0.63 (0.45–0.72)	0.64 (0.44–0.78)	0.83 (0.68–0.94)	0.72 (0.66–0.78)	0.70 (0.62–0.82)	0.69 (0.62–0.85)	0.76 (0.69–0.85)	0.71 (0.63–0.78)	0.76 (0.67–0.82)	0.81 (0.74–0.85)
EQ-5D	0.72 (0.68–0.76)	0.79 (0.73–0.85)	0.63 (0.48–0.71)	0.65 (0.44–0.78)	0.75 (0.61–0.81)	0.75 (0.67–0.78)	0.67 (0.61–0.73)	0.71 (0.63–0.86)	0.68 (0.61–0.73)	0.72 (0.64–0.78)	0.75 (0.67–0.81)	0.80 (0.73–0.84)
HUI	0.64 (0.54–0.67)	0.87 (0.85–0.89)	0.59 (0.44–0.68)	0.64 (0.34–0.71)	NR	NR	NR	NR	0.77 (0.70–0.82)	NR	0.76 (0.64–0.81)	NR
SF-6D	0.78 (0.73–0.83)	0.78 (0.74–0.80)	0.66 (0.64–0.69)	0.64 (0.63–0.67)	0.68 (0.68–0.68)	0.68 (0.67–0.68)	0.65 (0.64–0.71)	NR	0.72 (0.70–0.74)	0.64 (0.63–0.66)	0.65 (0.62–0.69)	0.81 (0.78–0.83)
SG	0.59 (0.50–0.68)	0.45 (0.45–0.45)	0.66 (0.45–0.86)	0.50 (0.08–0.65)	0.94 (0.88–0.96)	0.89 (0.89–0.89)	0.93 (0.73–0.97)	NR	0.93 (0.89–0.96)	NR	0.93 (0.92–0.98)	NR
TTO	0.87 (0.75–0.90)	0.49 (0.47–0.51)	0.55 (0.32–0.85)	0.33 (0.33–0.52)	1.00 (0.98–1.00)	0.52 (0.52–0.52)	0.82 (0.72–0.86)	0.56 (0.56–0.56)	0.84 (0.74–0.90)	NR	0.85 (0.70–0.94)	NR
Others^b^	0.64 (0.61–0.98)	0.90 (0.90–0.91)	0.48 (0.47–0.66)	0.68 (0.64–0.73)	0.78 (0.68–0.86)	0.75 (0.70–0.80)	0.90 (0.88–0.96)	NR	0.78 (0.73–0.80)	NR	0.80 (0.80–0.80)	0.89 (0.86–0.90)

^a^Four studies reported utilities for unstable angina, whereas the majority of studies in this group did not specify the type of angina

^b^Other methods include Quality of Well-Being, Utility Based Quality of life-Heart questionnaire, Assessment of Quality of Life, and Preference-based Stroke Index

*Abbreviations: EQ-5D* EuroQol Five Dimensions Questionnaire, *HUI* Health Utilities Index, *IQR* Interquartile range, *MI* Myocardial infarction, *PAD* Peripheral artery disease, *NR* Not reported, *SF-6D* Short-Form Six Dimensions, *SG* Standard gamble, *TTO* Time trade-off

**Fig. 3:**
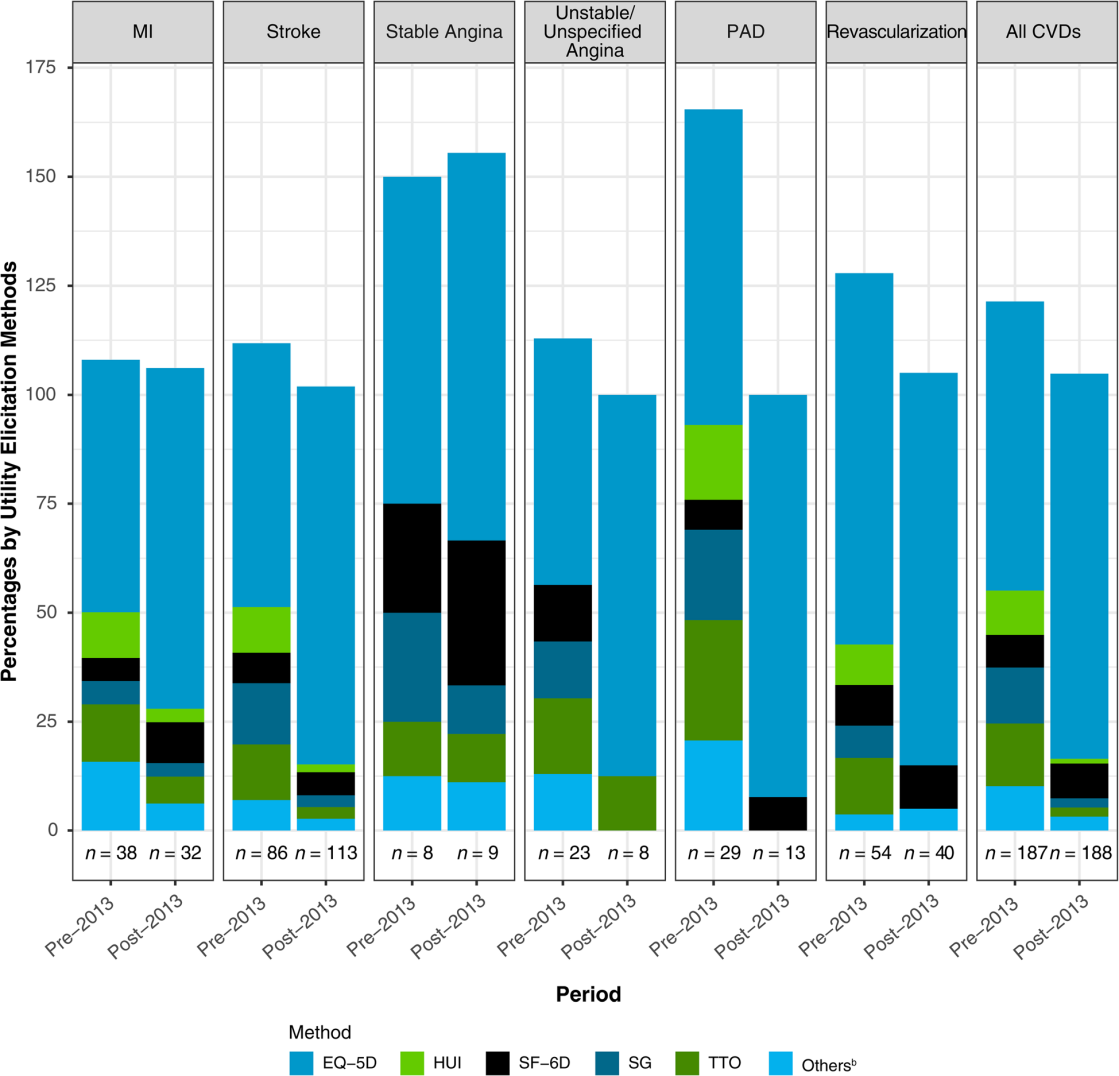
Proportion^a^ of utility values by method of elicitation**.**^a^Percentages may add to > 100%, as some studies reported utility values using more than 1 method of elicitation. ^b^Other methods include Quality of Well-Being, Utility Based Quality of life-Heart questionnaire, Assessment of Quality of Life, and Preference-based Stroke Index. *Abbreviations: CVD* cardiovascular disease, *EQ-5D* EuroQol Five Dimensions Questionnaire, *HUI* Health Utilities Index, *MI* myocardial infarction, *PAD* peripheral artery disease, *SF-6D* Short-Form Six Dimensions, *SG* standard gamble, *TTO* time trade-off

These findings varied by method of utility elicitation (Fig. 3). Across CV health states, utilities measured by the EQ-5D and the HUI typically improved over time when comparing studies published before 2013 to those published after 2013, whereas utilities measured by the direct methods (SG and TTO) consistently worsened over time. However, the HUI, SG, and TTO were only used by a limited number of studies within the last 6 years.

### Trends in utility values over time by method of utility elicitation

When comparing direct elicitation methods with indirect elicitation methods across CV health states, direct methods yielded, on average, the highest utilities within studies published prior to 2013; in contrast, direct methods yielded the lowest utility value estimates among studies published after 2013. This was largely driven by a reduction in the average utilities generated from direct methods over time (pre-2013 vs post-2013, respectively: 0.85 vs 0.50), as the change in utilities generated via indirect methods was less extreme (0.69 vs 0.73).

### Trends in utility over time by methodologies used by CV health state

Table 3 displays the characteristics of the included studies. The usage of the SG and TTO methods decreased substantially in the last 6 years across all CV health states (pre-2013 vs post-2013, respectively: SG, 12.8% vs 2.1%; TTO, 14.4% vs 2.1%). The EQ-5D was the most common utility elicitation method in both time periods, followed by the SF-6D. There was a notable increase in the use of the EQ-5D for all CV health states (pre-2013 vs post-2013, respectively: 66.3% vs 88.3%) in studies that were published in the last 6 years compared with studies published prior to 2013. Use of the HUI decreased substantially in the last 6 years across the CV health states (pre-2013 vs post-2013, respectively: HUI, 10.2% vs 1.1%) when compared to studies published before 2013. Of note, the HUI was not used among studies reporting utilities for angina in either time period. Use of the SF-6D showed a slight increase over the last 6 years (pre-2013 vs post-2013, respectively: 7.5% vs 8.0%), which was driven primarily by its increased incorporation for estimating utilities for MI, stable angina, PAD, and revascularization, as its use has actually decreased for stroke and unstable/unspecified angina.

**Table 3:** Characteristics of the 375 included studies^a^

	MI		Stroke		Stable angina		Unstable/unspecified angina		PAD		Revascularization		Total (across all CVDs)	
Measure	Pre-2013 N (%)	Post-2013 N (%)	Pre-2013 N (%)	Post-2013 N (%)	Pre-2013 N (%)	Post-2013 N (%)	Pre-2013 N (%)	Post-2013 N (%)	Pre-2013 N (%)	Post-2013 N (%)	Pre-2013 N (%)	Post-2013 N (%)	Pre-2013 N (%)	Post-2013 N (%)
n	38	32	86	113	8	9	23	8	29	13	54	40	187	188
**Method of elicitation**														
EQ-5D	22 (57.9)	25 (78.1)	52 (60.5)	98 (86.7)	6 (75.0)	8 (88.9)	13 (56.5)	7 (87.5)	21 (72.4)	12 (92.3)	46 (85.2)	36 (90.0)	124 (66.3)	166 (88.3)
HUI	4 (10.5)	1 (3.1)	9 (10.5)	2 (1.8)	0 (0)	0 (0)	0 (0)	0 (0)	5 (17.2)	0 (0)	5 (9.3)	0 (0)	19 (10.2)	2 (1.1)
SF-6D	2 (5.3)	3 (9.4)	6 (7.0)	6 (5.3)	2 (25.0)	3 (33.3)	3 (13.0)	0 (0)	2 (6.9)	1 (7.7)	5 (9.3)	4 (10.0)	14 (7.5)	15 (8.0)
SG	2 (5.3)	1 (3.1)	12 (14.0)	3 (2.7)	2 (25.0)	1 (11.1)	3 (13.0)	0 (0)	6 (20.7)	0 (0)	4 (7.4)	0 (0)	24 (12.8)	4 (2.1)
TTO	5 (13.2)	2 (6.2)	11 (12.8)	3 (2.7)	1 (12.5)	1 (11.1)	4 (17.4)	1 (12.5)	8 (27.6)	0 (0)	7 (13.0)	0 (0)	27 (14.4)	4 (2.1)
Others^b^	6 (15.8)	2 (6.2)	6 (7.0)	3 (2.7)	1 (12.5)	1 (11.1)	3 (13.0)	0 (0)	6 (20.7)	0 (0)	2 (3.7)	2 (5.0)	19 (10.2)	6 (3.2)
**Type of respondent**														
Patients	37 (97.4)	30 (93.8)	84 (97.7)	107 (94.7)	7 (87.5)	8 (88.9)	22 (95.7)	6 (75.0)	29 (100.0)	13 (100.0)	54 (100.0)	40 (100.0)	183 (97.9)	181 (96.3)
General population	1 (2.6)	2 (6.2)	3 (3.5)	5 (4.4)	1 (12.5)	1 (11.1)	1 (4.3)	2 (25.0)	0 (0)	0 (0)	0 (0)	0 (0)	5 (2.7)	6 (3.2)
Caregivers	0 (0)	0 (0)	3 (3.5)	2 (1.8)	0 (0)	0 (0)	0 (0)	0 (0)	0 (0)	0 (0)	0 (0)	0 (0)	3 (1.6)	2 (1.1)
Mixed	0 (0)	0 (0)	3 (3.5)	2 (1.8)	0 (0)	0 (0)	0 (0)	0 (0)	0 (0)	0 (0)	0 (0)	0 (0)	3 (1.6)	2 (1.1)
**Study design**														
Trial	9 (23.7)	14 (43.8)	10 (11.6)	37 (32.7)	2 (25.0)	3 (33.3)	2 (8.7)	2 (25.0)	12 (41.4)	4 (30.8)	18 (33.3)	20 (50.0)	42 (22.5)	70 (37.2)
Survey	16 (42.1)	10 (31.2)	34 (39.5)	28 (24.8)	4 (50.0)	3 (33.3)	16 (69.6)	4 (50.0)	5 (17.2)	4 (30.8)	6 (11.1)	4 (10.0)	56 (29.9)	39 (20.7)
Prospective cohort	9 (23.7)	4 (12.5)	31 (36.0)	37 (32.7)	1 (12.5)	2 (22.2)	2 (8.7)	1 (12.5)	7 (24.1)	4 (30.8)	25 (46.3)	12 (30.0)	67 (35.8)	59 (31.4)
Retrospective cohort	1 (2.6)	4 (12.5)	5 (5.8)	8 (7.1)	0 (0)	0 (0)	0 (0)	1 (12.5)	5 (17.2)	1 (7.7)	4 (7.4)	5 (12.5)	13 (7.0)	17 (9.0)
Others (analysis, NR)	3 (7.9)	0 (0)	6 (7.0)	3 (2.7)	1 (12.5)	1 (11.1)	3 (13.0)	0 (0)	1 (3.4)	0 (0)	1 (1.9)	0 (0)	10 (5.3)	4 (2.1)
**Geographic region**														
Europe	16 (42.1)	15 (46.9)	40 (46.5)	51 (45.1)	4 (50.0)	4 (44.4)	9 (39.1)	2 (25.0)	22 (75.9)	7 (53.8)	33 (61.1)	15 (37.5)	92 (49.2)	83 (44.1)
US and Canada	14 (36.8)	4 (12.5)	27 (31.4)	17 (15.0)	1 (12.5)	1 (11.1)	10 (43.5)	2 (25.0)	5 (17.2)	3 (23.1)	8 (14.8)	7 (17.5)	53 (28.3)	27 (14.4)
Asia	1 (2.6)	5 (15.6)	6 (7.0)	30 (26.5)	2 (25.0)	2 (22.2)	1 (4.3)	3 (37.5)	0 (0)	2 (15.4)	3 (5.6)	5 (12.5)	11 (5.9)	39 (20.7)
Others^c^	7 (18.4)	8 (25.0)	13 (15.1)	21 (18.6)	1 (12.5)	2 (22.2)	4 (17.4)	1 (12.5)	2 (6.9)	2 (15.4)	12 (22.2)	13 (32.5)	33 (17.6)	46 (24.5)

^a^Percentages may add to > 100% for utility method, respondent, or geographic region, as some studies reported utility values in more than 1 category (ie, a study that compared EQ-5D to SF-6D would count in both categories)

^b^Other methods include Quality of Well-Being, Utility Based Quality of life-Heart questionnaire, Assessment of Quality of Life, and Preference-based Stroke Index

^c^Others include studies conducted in Australia, Brazil, Israel, Mexico, New Zealand, Nigeria, and Zimbabwe, as well as studies conducted in multiple regions or those that did not report geography

*Abbreviations: CVD* Cardiovascular disease, *EQ-5D* EuroQol Five Dimensions Questionnaire, *HUI* Health Utilities Index, *MI* Myocardial infarction, *NR* Not reported, *PAD* Peripheral artery disease, *SF-6D* Short-Form Six Dimensions, *SG* Standard gamble, *TTO* Time trade-off, *US* United States

The increase in use of the EQ-5D coincides with an increase in trials generating utility data (Supplementary Fig. 1). The proportion of utility estimates coming from trials increased from 22.5% (pre-2013) to 37.2% (post-2013) across all CV health states. Prior to 2013, prospective cohort studies were the most commonly used study design to derive utilities from CV health states (35.8%), whereas over the last 6 years this decreased to 31.4%. Trials were more likely to measure utilities via the EQ-5D, with 31 of 42 (73.8%) trials and 93 of 145 (64.1%) other study designs publishing CV utility values prior to 2013 using the EQ-5D. The increase in use of the EQ-5D can be attributed not only to the increase in the proportion of trials publishing utilities, but also that trials currently use the EQ-5D more often, with 64 of 70 (91.4%) trials publishing CV utility values in the last 6 years using the EQ-5D. An increase in the use of the EQ-5D among surveys and prospective cohorts was also observed in the last 6 years, with 25 of 39 (64.1%) surveys and 54 of 59 (91.5%) prospective cohorts publishing CV utility values.

The use of direct methods declined specifically for studies eliciting utilities from patients, but otherwise remained steady (Supplementary Fig. 2). The proportion of studies using indirect methods that evaluated respondents other than patients (general population, caregivers, or mixed) increased slightly from 1.5% among studies published before 2013 to 2.8% among more recent studies. However, it should be noted that only a few studies elicited values from respondents other than patients. Across CV health states, most studies that reported utilities derived them from patients in both time periods (pre-2013 vs post-2013, respectively: 183 of 187 [97.9%] vs 181 of 188 [96.3%]). The proportion of utility values derived from general-population studies remained stable over this period (pre-2013 vs post-2013, respectively: 5 of 187 [2.7%] vs 6 of 188 [3.2%]). Since the proportion of studies using direct methods has decreased, and the proportion of studies eliciting utilities from general-population respondents has slightly increased, it follows that the proportion of direct measures evaluating general-population respondents increased from pre-2013 (5 of 42 [11.9%]) to post-2013 (4 of 8 [50.0%]) among all CV health states. Only a few studies featured utility values derived from caregivers or mixed respondents, all of which estimated utilities for stroke as the health state.

### Trends in utility over time by geographical region

Across CV health states and in both time periods, most studies were conducted in Europe, followed by the United States (US) and Canada (Supplementary Fig. 3). There was a decrease in studies conducted in the US and Canada in the last 6 years (pre-2013 vs post-2013, respectively: 28.3% vs 14.4%). In contrast, more utility studies emerged from Asia. Among the studies conducted in Asia, the vast majority of data reported were for stroke, and there were a limited number of studies reporting utility values for the other CV health states.

## Discussion

The goal of this SLR was to update and expand upon the review conducted by Smith et al. [12] to identify the most recent utility values for stroke, MI, angina, PAD, and revascularization and to gain insight into changing trends in utility values over time and corresponding elicitation methods. The results of the SLR are summarized qualitatively, to depict the variation in utility values observed across studies in this broad SLR. The decision not to conduct a meta-analysis was further confirmed by a 2015 review of utility value meta-analyses, which found substantial differences when direct vs indirect methods were compared, and noted that meta-techniques may not be appropriate given substantial heterogeneity among utility methods [18]. In another systematic review on the EQ-5D utility values in CVD, the authors attempted to conduct a meta-analysis but deemed it was inappropriate to further estimate pooled utility scores via meta-analytic techniques due to the substantial observed heterogeneity with respect to both study design and patient characteristics. Given this observed heterogeneity, effect sizes obtained via meta-analytic techniques are not generalizable to other methods or health states [19].

This SLR reports utility values consistent with values used in several large-scale economic evaluations in CVD [20, 21], in particular for MI and stroke. For example, in the study conducted by Ara et al. [20], the authors used the Health Surveys for England (HSEs) conducted in 2003 and 2006 to elicit EQ-5D scores for stroke, heart attack, and angina; the mean EQ-5D score for patients with heart attack was 0.74, which was in line with the median (IQR) utility value pre-2013 for MI in the current SLR (0.72 [0.68–0.76]). The mean EQ-5D utility value for individuals with stroke was 0.66, which aligns with the median (IQR) utility value post-2013 for stroke observed in the current SLR (0.65 [0.44–0.78]). In our study, widely varying utility values were observed for stroke. The observed heterogeneity suggests that it may be difficult to assess utilities for stroke.

The mean utility value for angina (0.692) in the Ara et al. [20] paper was slightly lower compared with the pre-2013 median (IQR) utility values for angina (stable angina, 0.83 [0.68–0.94]; unstable/undefined angina, 0.70 [0.62–0.82]), but similar to post-2013 values (stable angina, 0.72 [0.66–0.78]; unstable/undefined angina, 0.69 [0.62–0.85]) in the current review. The study conducted by Ara et al. did not report utility values on PAD or revascularization.

In comparing the 187 CV utility studies published during or before 2012 with the 188 published during or after 2013, we observed changes in recent years with respect to the actual values being published in CV utility studies; average utility values for MI, stroke, PAD, and revascularization increased over this period, whereas utilities for angina declined. This likely represents changes in the types of populations and health states being measured. Improvements in healthcare over time may have also contributed to the observed changes. Disease characteristics, disease severity, and duration of disease all contribute to substantial variation in utility scores [12, 22]. As we did not control for sample characteristics, the higher utility estimates observed for several instruments could have been influenced by the population under evaluation rather than the specific utility method. These changes in recent years underscore the necessity of selecting utility values that precisely represent the health state of interest for a cost-utility analysis.

However, the observed changes in CV values, particularly the increases observed for MI and stroke, may be confounded by changes in the methods used. This review found that the EQ-5D is the most common measure across types of CV health states, its use is increasing, and it yielded higher utilities than direct methods in the last 6 years. In the last 6 years, the average values for indirect measures have risen, whereas the average values for direct methods have declined. This increase in the use of the EQ-5D appears to be related to the general increase in trials estimating utility values; however, even among trials, the EQ-5D was utilized more frequently in the last 6 years. The National Institute for Health and Care Excellence (NICE) and other payers, such as the Scottish Medicines Consortium (SMC), recommended the EQ-5D as the preferred utility in the reference case for cost-utility analyses in 2004 (to encourage comparability across studies) [23] and clarified recommendations in 2013 [24], and it is possible that these trends reflect increasing uptake of those recommendations in the last 6 years. It is also likely that trials are measuring utility values more often, as the need for cost-utility assessment, and consequently utility values representing the precise patient population in question, has grown in the current health reimbursement market. Our review also observed a substantial increase in the number of CV utility studies conducted in Asia. This likely reflects the growing HTA trend in this part of the world [25, 26].

Furthermore, our review observed that the implementation of direct utility elicitation methods has declined substantially in CVDs in recent years. This could be due to the ease of implementation and lower cost of a standardized questionnaire, such as the EQ-5D, compared with direct methods, which often require bespoke design. In addition, it is likely that investigators are also relying more frequently on the EQ-5D as they have become increasingly comfortable with the validity of the measure. However, given that indirect methods represent an estimation of utility values and do not measure the patient preference, direct methods should still be considered a valuable tool. We did not include VAS utility measures in our SLR because of their potentially limited use for measuring preferences for health states [15]. Moreover, others have raised concerns regarding the validity of VAS for this purpose [27–29].

The differences in utility values across methods observed in our review is well documented in the literature [30–35]. However, the relationship between direct and indirect utility measures has not been as thoroughly documented. There is a widely held impression among health economists that direct methods tend to yield higher utilities (reflecting better reported health) for given health states compared with indirect methods, regardless of the type of direct or indirect method used (eg, TTO vs SF-6D or EQ-5D vs SF-6D) [36–42]. Different methods of utility elicitation can result in varying scores, even for the same patient population assessed. For example, Hallan et al. [43] used both SG and TTO methods and found significantly higher scores for both minor and major stroke health states using SG compared with TTO assessments. Utility scales also vary in sensitivity, which may further hinder comparisons of utility values across measures. For example, the EQ-5D index score has been shown to have a ceiling effect, and the SF-6D has been observed to have a floor effect [31, 44, 45]. However, the EQ-5D scale has been reported to be more sensitive than the SF-6D in monitoring values for HRQoL, particularly at the lower end of the scale for patients with chronic obstructive airways disease, osteoarthritis, irritable bowel syndrome, lower back pain, or leg ulcers, and for postmenopausal women and healthy elderly individuals (aged 75+ years) [31]; this trend was also observed in the current SLR, although this is not usually the case for CVD. In addition, the HUI focuses on physical and emotional health and does not include questions on social functioning or satisfaction [46].

## Conclusion

This review found that health utility values for MI, stroke, angina, PAD, and revascularization have changed substantially when comparing different time periods (pre-2013 vs post-2013), likely due to changes in the types of studies being conducted (increase in trials eliciting utilities) and the patient populations being evaluated (in particular, changes in disease severity and duration of disease). Changing utility methods may also partially explain the observed changes in utility values. The EQ-5D has been used more frequently, with an increasing number of trials using this measure. Additionally, an increasing number of studies in Asia estimating CV utilities has been observed. With varying values observed across utility methods used and populations, care should be taken when choosing utility values to use in economic evaluations of new technologies. Future analyses that assess changes in utilities by duration of disease and/or treatment could be useful to identify any trends for patients with early vs late stage disease and help inform the choice of utility values for use in economic evaluations of new cardiovascular technologies.

## Supplementary information

**Additional file 1.** File format including the correct file extension (including name and a URL of an appropriate viewer if format is unusual). Supplementary Material. Pages: 12. Figures: 3. Tables: 5.

## Data Availability

Data sharing is not applicable to this article as no datasets were generated or analyzed during the current study.

## Abbreviations

CEA: Cost-effectiveness analysis
CV: Cardiovascular
CVD: Cardiovascular disease
EQ-5D: EuroQol Five Dimensions Questionnaire
HRQoL: Health-related quality of life
HSE: Health Surveys for England
HTA: Health technology assessment
HTAD: Health Technology Assessment Database
HUI: Health utilities index
IQR: Interquartile range
MI: Myocardial infarction
MID: Minimally important difference
NICE: National Institute for Health and Care Excellence
PAD: Peripheral artery disease
PICOS: Participants, interventions, comparators, outcomes, and study design
PRISMA: Preferred Reporting Items for Systematic Reviews and Meta-Analyses
QALY: Quality-adjusted life-year
SF-6D: Short-Form Six Dimensions
SG: Standard gamble
SLR: Systematic literature review
SMC: Scottish Medicines Consortium
TTO: Time trade-off
US: United States
