# Supplementary material for: Utility value estimates in cardiovascular disease and the effect of changing elicitation methods: a systematic literature review
Source: Health Qual Life Outcomes. 2020 Jul 27;18:251. doi: 10.1186/s12955-020-01407-y (PMC7385861; doi:10.1186/s12955-020-01407-y)
Supplement: Supplementary file 1 — Additional file 1. File format including the correct file extension (including name and a URL of an appropriate viewer if format is unusual). Supplementary Material. Pages: 12. Figures: 3. Tables: 5. [file 12955_2020_1407_MOESM1_ESM.docx]

SUPPLEMENTARY MATERIAL

Title: Utility value estimates in cardiovascular disease and the effect of changing elicitation methods: a systematic literature review

**Authors:** Marissa Blieden Betts, MS^1*^, Pratik Rane, MBA, PhD^2^, Evelien Bergrath, MSc^1^, Madhura Chitnis, PhD^1^, Mohit Kumar Bhutani, M.Pharm^3^, Claudia Gulea, MSc^4^, Yi Qian, PhD^5^, Guillermo Villa, PhD^6^

**Affiliations:**

^1^Evidence Synthesis, Modeling & Communication, Evidera, Waltham, MA, USA

^2^Amgen Inc, Global Health Economics, Thousand Oaks, CA, USA

^3^BresMed Health Solutions Ltd., Gurugram, Haryana, India

^4^Evidence Synthesis, Modeling & Communication, Evidera, London, UK

^5^Amgen Inc, Intercontinental HQ-Value, Access & Policy, Thousand Oaks, CA, USA

^6^Amgen (Europe) GmbH, Global Health Economics, Zug, Switzerland

Supplementary Table 1 Embase search terms

| **Disease string** | |
| --- | --- |
|  | attack:ab,ti AND (ischemic:ab,ti OR ischaemic:ab,ti) OR (cerebrovascular:ab,ti AND (event*:ab,ti OR infarction*:ab,ti OR accident*:ab,ti)) OR (infarction*:ab,ti AND (myocardial:ab,ti OR cardiac:ab,ti)) OR stroke*:ab,ti OR stemi:ab,ti OR nstemi:ab,ti OR 'heart attack':ab,ti OR 'revascularization':ab,ti OR 'revascularisation':ab,ti OR ptca:ab,ti OR pci:ab,ti OR 're vascularisation':ab,ti OR 're vascularization':ab,ti OR 'vascular bypass':ab,ti OR angioplast*:ab,ti OR 'coronary artery bypass surgery' OR 'percutaneous coronary intervention' OR 'transluminal coronary angioplasty' OR ((cardiovascular:ab,ti OR cardiac:ab,ti OR coronary:ab,ti) AND (death*:ab,ti OR mortal*:ab,ti)) OR angina*:ab,ti OR angioplast*:ab,ti OR 'coronary bypass':ab,ti OR stent*:ab,ti OR (percutaneous:ab,ti AND coronary:ab,ti) OR cabg:ab,ti OR ((cardiac:ab,ti OR coronary:ab,ti OR cardiovascular:ab,ti) AND catheterize*:ab,ti) OR apheresis:ab,ti OR aphereses:ab,ti OR phereses:ab,ti OR pheresis:ab,ti OR 'blood component removals':ab,ti OR ('component removal':ab,ti AND blood:ab,ti) OR ('component removals':ab,ti AND blood:ab,ti) OR 'heart failure':ab,ti OR 'cardiac failure':ab,ti OR 'myocardial failure':ab,ti OR 'heart decompensation':ab,ti OR 'new york heart association':ab,ti OR nyha:ab,ti OR 'peripheral arterial disease' OR pad:ab,ti OR (narrow* NEAR/4 arter*) OR 'peripheral occlusive artery disease' OR 'peripheral occlusive artery disease':ab,ti |
| **Utilities string** | |
|  | 'health utility':ab,ti OR 'health utilities':ab,ti OR 'health state utility':ab,ti AND 'health state utilities':ab,ti OR 'utility score':ab,ti OR 'utility value':ab,ti OR 'standard gamble':ab,ti OR 'time trade-off':ab,ti OR 'time tradeoff':ab,ti OR 'time trade off':ab,ti OR preference*:ab,ti OR 'eq 5d':ab,ti OR eq5d:ab,ti OR 'utility assessment':ab,ti OR hui:ab,ti OR 'short form 6d':ab,ti OR 'short-form 6d':ab,ti OR sf6d:ab,ti OR 'sf 6d':ab,ti OR 'willingness to pay':ab,ti OR 'willingness-to-pay':ab,ti OR 'quality adjusted life year':ab,ti OR 'quality-adjusted life year':ab,ti OR 'cost utility analysis':ab,ti OR 'cost-utility analysis':ab,ti OR 'health technology assessment':ab,ti OR euroqol:ab,ti |

Supplementary Table 2 PubMed search terms

| **Disease string** | |
| --- | --- |
|  | (((attack AND (ischemic OR ischaemic)) OR (cerebrovascular AND (event* OR infarction* OR accident*)) OR (infarction* AND (myocardial OR cardiac)) OR stroke* OR "STEMI" OR "NSTEMI" OR "heart attack" OR "revascularization" OR "revascularisation" OR "PTCA" OR "PCI" OR "re vascularization" OR "re vascularization" OR "vascular bypass" OR angioplast* OR "coronary artery bypass surgery" OR "percutaneous coronary intervention" OR "transluminal coronary angioplasty" OR (("cardiovascular" OR "cardiac" OR "coronary") AND (death* OR mortal*)) OR angina* OR angioplast* OR "coronary bypass" OR stent* OR ("percutaneous" AND "coronary") OR "CABG" OR ((cardiac OR coronary OR cardiovascular) AND catheterize*)) OR ((apheresis OR aphereses OR phereses OR pheresis OR "Blood Component Removals" OR ("Component Removal" AND Blood) OR ("Component Removals" AND Blood) OR (Removal AND "Blood Component") OR (Removals AND "Blood Component")) AND (LDL* OR (Lipoprotein* AND low-density))) OR ("heart failure" OR "cardiac failure" OR "myocardial failure" OR "heart decompensation" OR "New York Heart Association" OR NYHA))) OR "peripheral arterial disease" OR pad OR (narrow* AND arter*) OR "peripheral occlusive artery disease" OR "peripheral occlusive artery disease") |
| **Utilities string** | |
|  | (("health utility" OR "health utilities" OR "health state utility" OR "health state utilities" OR "utility score" OR "utility value" OR "Standard gamble" OR "time trade-off" OR "time tradeoff" OR "time trade off" OR preference* OR eq-5d OR EQ5D OR "utility assessment" OR hui OR "short form 6d" OR "short-form 6d" OR sf6d OR sf-6d OR "sf 6d" OR "willingness to pay" OR "willingness-to-pay" OR "quality adjusted life year" OR "quality-adjusted life year" OR "cost utility analysis" OR "cost-utility analysis" OR "health technology assessment" OR EuroQol)) |

Supplementary Table 3 HTAD search terms

| **Disease string** | |
| --- | --- |
| **1**. | "revascularization":ab,ti,kw or "revascularisation":ab,ti,kw or "re vascularisation":ab,ti,kw or "re vascularization":ab,ti,kw or "vascular bypass":ab,ti,kw or angioplast*:ab,ti,kw or "coronary artery bypass surgery" or "percutaneous coronary intervention" or "transluminal coronary angioplasty" or "peripheral arterial disease" or pad:ab,ti,kw or (narrow* near/4 arter*) or "peripheral occlusive artery disease" or "peripheral occlusive artery disease":ab,ti,kw or attack:ab,ti,kw and (ischemic:ab,ti,kw or ischaemic:ab,ti,kw) or (cerebrovascular:ab,ti,kw and (event*:ab,ti,kw or infarction*:ab,ti,kw or accident*:ab,ti,kw)) or (infarction*:ab,ti,kw and (myocardial:ab,ti,kw or cardiac:ab,ti,kw)) or stroke*:ab,ti,kw or stemi:ab,ti,kw or nstemi:ab,ti,kw or 'heart attack':ab,ti,kw or revascularization:ab,ti,kw or revascularisation:ab,ti,kw or ptca:ab,ti,kw or pci:ab,ti,kw or (cardiovascular:ab,ti,kw or cardiac:ab,ti,kw or coronary:ab,ti,kw) and (death*:ab,ti,kw or mortal*:ab,ti,kw) or angina*:ab,ti,kw or angioplast*:ab,ti,kw or "coronary bypass":ab,ti,kw or stent*:ab,ti,kw or percutaneous:ab,ti,kw and coronary:ab,ti,kw or cabg:ab,ti,kw or apheresis:ab,ti,kw or aphereses:ab,ti,kw or phereses:ab,ti,kw or pheresis:ab,ti,kw or "blood component removals":ab,ti,kw or (cardiac:ab,ti,kw or coronary:ab,ti,kw or cardiovascular:ab,ti,kw) and catheterize*:ab,ti,kw or "component removal":ab,ti,kw and blood:ab,ti,kw or "component removals":ab,ti,kw and blood:ab,ti,kw or "heart failure":ab,ti,kw or "cardiac failure":ab,ti,kw or "myocardial failure":ab,ti,kw or "heart decompensation":ab,ti,kw or "heart decompenzation":ab,ti,kw or "New York heart association":ab,ti,kw or NYHA:ab,ti,kw |
| **Utilities string** | |
| **2.** | (("health utility" OR "health utilities" OR "health state utility" OR "health state utilities" OR "utility score" OR "utility value" OR "Standard gamble" OR "time trade-off" OR "time tradeoff" OR "time trade off" OR preference* OR eq-5d OR EQ5D OR "utility assessment" OR hui OR "short form 6d" OR "short-form 6d" OR sf6d OR sf-6d OR "sf 6d" OR "willingness to pay" OR "willingness-to-pay" OR "quality adjusted life year" OR "quality-adjusted life year" OR "cost utility analysis" OR "cost-utility analysis" OR "health technology assessment" OR EuroQol)) |

*Abbreviation:* *HTAD* Health Technology Assessment Database

Supplementary Table 4 Gray literature sources

| Scientific Conferences |
| --- |
| - American College of Cardiology (ACC) - American Heart Association (AHA) - American Heart Association, Quality of Care and Outcomes Research Scientific Sessions (AHA QCOR) - European Atherosclerosis Society (EAS) - European Society of Cardiology (ESC) - International Society for Pharmacoeconomics and Outcomes Research (ISPOR) - Canadian Agency for Drugs and Technologies in Health (CADTH) - European Medicines Agency (EMA) - National Health Service Economic Evaluation Database (NHS EED) - National Institute for Health and Care Excellence (NICE) - Scottish Medicines Consortium (SMC) - United States (US) Food and Drug Administration (FDA) - The School of Health and Related Research (ScHARR) |

Supplementary Table 5 PICOS criteria for inclusion/exclusion of studies

|  | Inclusion | Exclusion |
| --- | --- | --- |
| **Patients** | Patients aged 18+ years with any of the following CV health states:   - MI - Stroke (ischemic, hemorrhagic, or TIA) - Angina (stable and unstable) - PAD - Revascularization (any-cause, including both peripheral and coronary) | Patients < 18 years old or without CV health states of interest |
| **Intervention/ Comparators** | Any treatment | N/A |
| **Outcomes** | Absolute health state utility value | - No health state utility value reported - Disutilities or utility multipliers |
| **Study Design** | Study generates the utility value (ie, “primary” utility values) | Study reports only previously published utility values (eg, literature reviews or economic models not reporting primary utility values) |

*Abbreviations:* *CV* cardiovascular, *MI* myocardial infarction, *N/A* not applicable, *PAD* peripheral artery disease, *PICOS* patients, intervention/comparators, outcomes, study design, *TIA* transient ischemic attack

**Supplementary Fig. 1** Proportion^*^ of utility values by study design


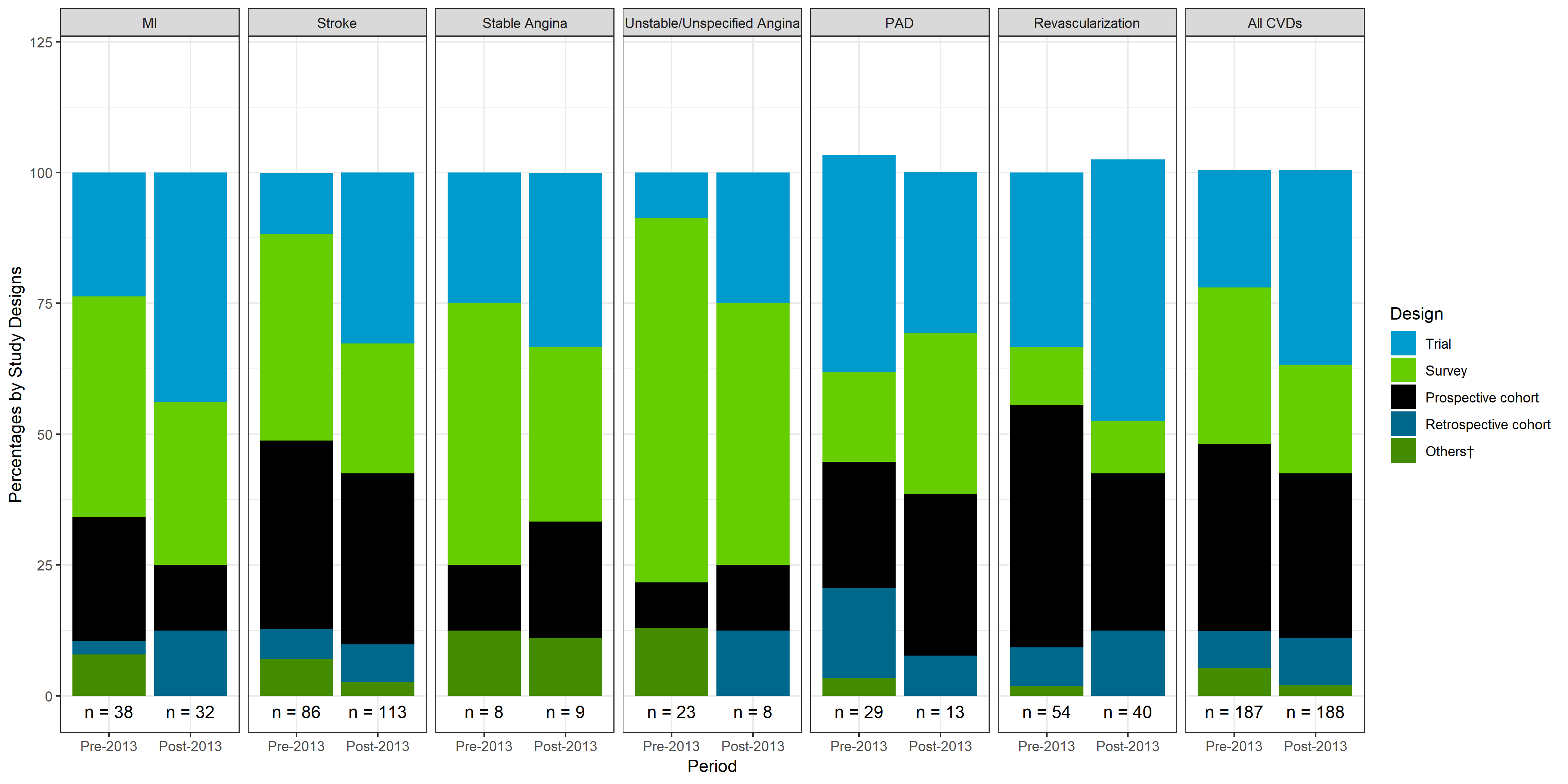


^*^Percentages may add to > 100%, as some publications reported utility values using more than 1 study design.

^†^Other includes analyses or studies for which the design is not reported.

*Abbreviations:* *CVD* cardiovascular disease, *MI* myocardial infarction, *PAD* peripheral artery disease

**Supplementary Fig. 2** Proportion^*^ of utility values by type of respondent


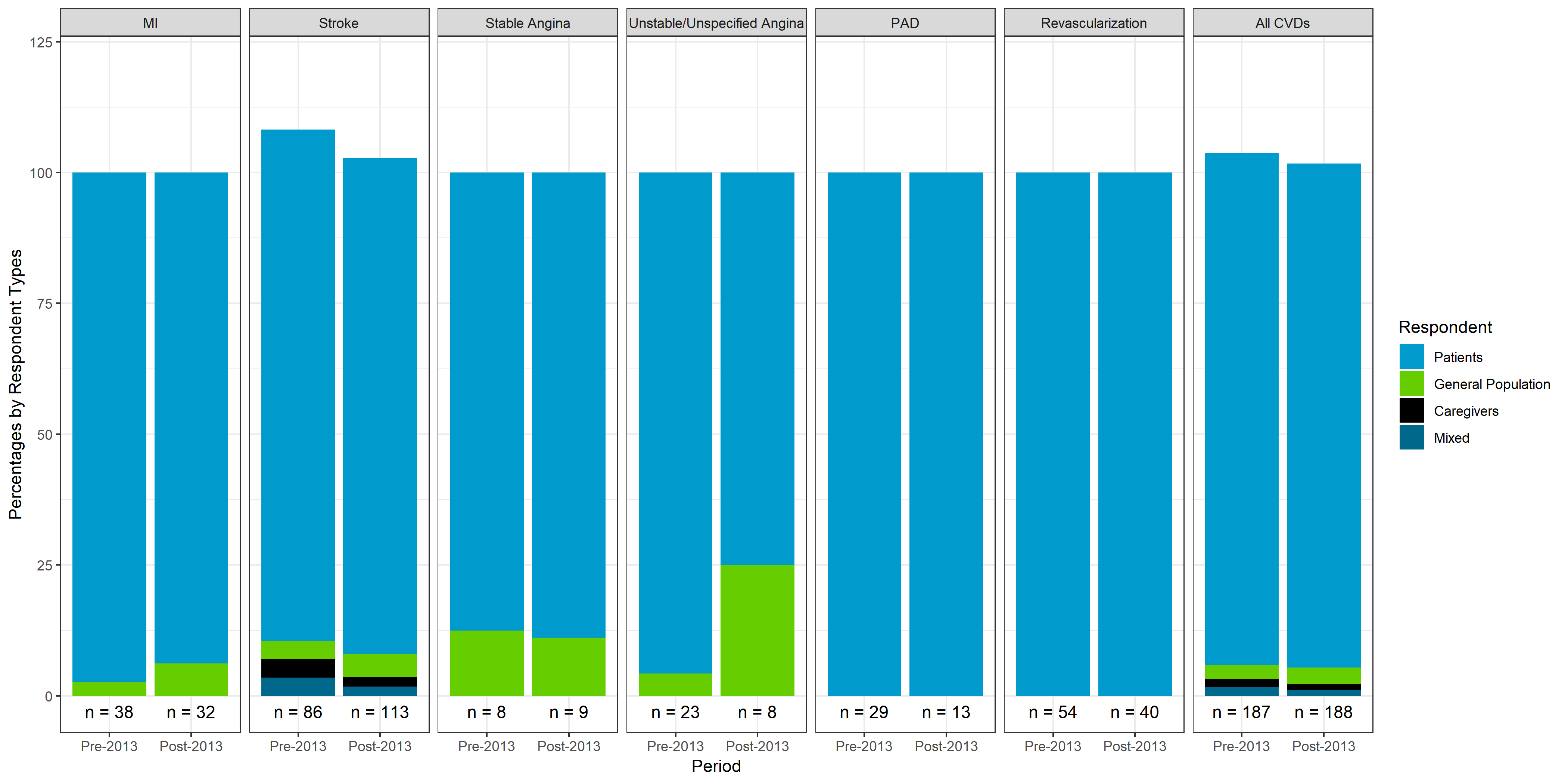


*Percentages may add to > 100%, as some studies reported utility values from more than 1 type of respondent.

*Abbreviations:* *CVD* cardiovascular disease, *MI* myocardial infarction, *PAD* peripheral artery disease

**Supplementary Fig. 3** Proportion* of utility values by geographical region


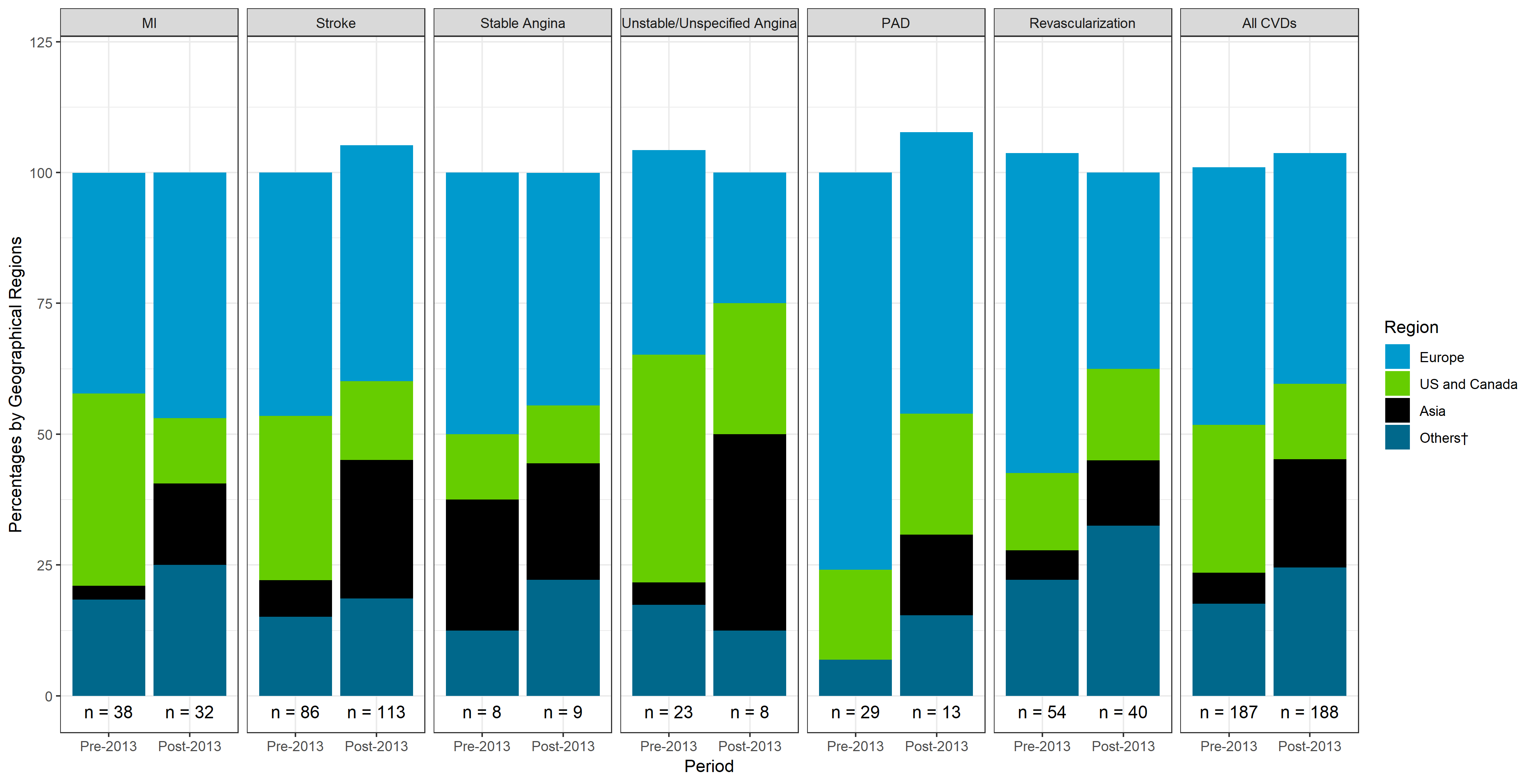


^*^Percentages may add to > 100%, as some studies reported utility values from more than 1 geographical region.

^†^Others include studies conducted in Australia, Brazil, Israel, Mexico, New Zealand, Nigeria, and Zimbabwe, as well as studies conducted in multiple regions or those that did not report geography.

*Abbreviations:* *CVD* cardiovascular disease, *MI* myocardial infarction, *PAD* peripheral artery disease, *US* United States
